# Epicardial coronary spasm due to endothelial dysfunction after spontaneous coronary artery dissection

**DOI:** 10.1007/s12471-019-01354-4

**Published:** 2019-11-27

**Authors:** V. E. Stegehuis, R. M. Dennert, T. P. van de Hoef, J. J. Piek

**Affiliations:** 1 Heart Centre, Department of Clinical and Experimental Cardiology, Amsterdam Cardiovascular Sciences, Amsterdam UMC, University of Amsterdam, Amsterdam, The Netherlands; 2 Department of Cardiology, Dr. Horacio E. Oduber Hospital, Oranjestad, Aruba

A 70-year-old woman underwent acetylcholine provocation testing for coronary spasm, due to typical angina symptoms and absence of obstructive coronary artery disease (CAD) as revealed by repeat coronary angiography (CAG). She had a previous conservatively treated spontaneous coronary artery dissection (SCAD).

SCAD is considered a rare non-atherosclerotic cause of acute coronary syndrome, treated predominantly conservatively, as revascularisation is associated with complications. The prevalence of SCAD is higher in women (88.5% in a recent study [1]). Moreover, women have a higher risk of symptoms and signs of myocardial ischaemia than men, but simultaneously have a 30–50% chance of having non-obstructive coronary artery disease (NOCAD) when undergoing CAG. Both SCAD and NOCAD are underdiagnosed, but awareness of these conditions is nowadays increasing [2]. The long-term effects of SCAD are largely unknown.

In the present case, coronary spasm occurred where previously a SCAD was documented (see Fig. 1). This illustrates that SCAD may cause endothelial dysfunction, resulting in angina symptoms. SCAD and NOCAD are characterised by a distinct risk profile, which is significantly different than that of conventional CAD [3, 4]. Vigilance for endothelial dysfunction causing angina symptoms is warranted.

**Fig. 1:**
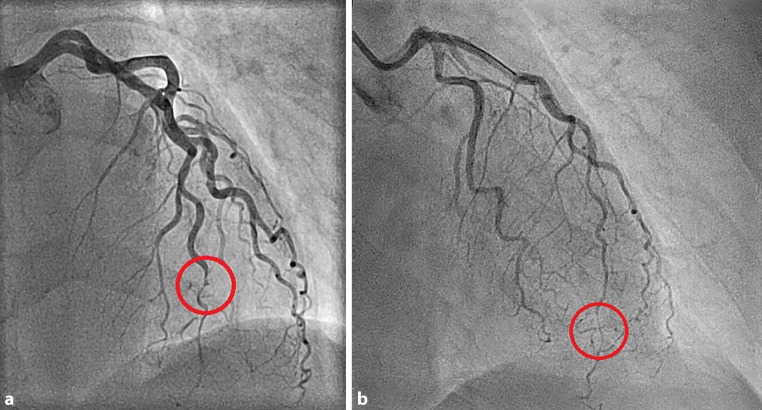
Angiography of the left anterior descending artery. **a** Initial angiogram in 2014 showing dissection of the distal left anterior descending (LAD) artery (indicated by the *red circle*). **b** Repeat angiogram in 2018. Coronary spasm of the distal LAD artery occurring mainly at the location of the healed spontaneous coronary artery dissection

## Caption Electronic Supplementary Material

1. Coronary angiography showing spontaneous coronary artery dissection (SCAD) of the distal left anterior descending artery (LAD) in 2014

2. Coronary angiography showing spontaneous coronary artery dissection of the distal LAD in 2014

3. Coronary angiography showing coronary spasm of the distal LAD in 2018, at the location where previously the SCAD was documented

4. Coronary angiography showing recovery of the coronary spasm of the distal LAD after intracoronary administration of nitroglycerin, at the location where previously the SCAD was documented
